# ﻿Inflorescences of *Fargesiaangustissima* T.P. Yi and *Yushaniapauciramificans* T.P. Yi (Poaceae, Bambusoideae) shed light on the taxonomy of the Sino-Himalayan alpine bamboos

**DOI:** 10.3897/phytokeys.215.94010

**Published:** 2022-12-09

**Authors:** Xia-Ying Ye, Zu-Chang Xu, Yue-Hong Cheng, Wei-Hua Wang, De-Zhu Li

**Affiliations:** 1 Agronomy and Life Science Department, Zhaotong University, Zhaotong, Yunnan 657000, China Kunming Institute of Botany, Chinese Academy of Sciences Yunnan China; 2 Germplasm Bank of Wild Species, Kunming Institute of Botany, Chinese Academy of Sciences, Kunming, Kunming, Yunnan 650201, China Zhaotong University Yunnan China; 3 Sichuan Wolong National Natural Reserve Administration, Aba, Sichuan, 623000, China Sichuan Wolong National Natural Reserve Administration Sichuan China

**Keywords:** *Borinda*, *Fargesia*, infloresence, reproductive characters, *Yushania*

## Abstract

The taxonomy of the Sino-Himalayan alpine bamboos is controversial due to their complex evolutionary history and further complicated by the scarcity of inflorescence. Here, we supplement the description of the inflorescence of *Fargesiaangustissima* T.P. Yi and *Yushaniapauciramificans* T.P. Yi, which shed light on the taxonomy of *Fargesia* Franchet, *Borinda* Stapleton and *Yushania* Keng. *F.angustissima* has compressed inflorescence unilateral stretching out from reduced spathe, showing a transitional state between species with condensed inflorescence embraced by spathe-like bracts and species with open inflorescence without bracts. Considering that extensive gene flow existed between several clades of *Fargesia* found in recent studies, a broadly-defined *Fargesia* s. l. should be adopted. Meanwhile, the inflorescence of *Y.pauciramificans* has typical characteristics of *Yushania*, such as axilla with tuberculate glands, rachilla internodes ciliate and cylindrical florets, supporting the delimitation of *Yushania*.

## ﻿Introduction

Although the taxonomy of bamboos has entered a new stage since the proliferation of molecular phylogenetic and phylogenomic studies (e.g., Bamboo Phylogeny Group 2012; Attigala et al. 2016; Zhang et al. 2020; Ye et al. 2021b), morphological characters play an important role in the naming and identification of the species. Reproductive characters are traditionally assumed to be critical in bamboo evolution and taxonomy, especially at the generic level (Li et al. 2006). However, due to variable blooming intervals, ranging from a couple of decades up to 120 years (Janzen 1976), many bamboo species were described without inflorescence information (Yi 1986, 1988a; Stapleton 1994; Keng and Wang 1996; Ohrnberger 1999; Li et al. 2006). This has caused the confusion in the definition of some genera, including some Sino-Himalayan alpine bamboos, e.g., *Fargesia* Franchet, *Yushania* Keng f. and *Borinda* Stapleton (Guo et al. 2002; Stapleton 2021).

*Fargesia* was delimited as having short-necked pachymorph rhizome with unicaespitose culms and compressed inflorescence subtended by several small or large spathes, while *Yushania* has long-necked rhizome with diffuse culms and open inflorescence without bracts (Li et al. 2006; Yi et al. 2008; Shi et al. 2022). *Borinda* was described as clumping temperate bamboos, similar to *Yushania* for its inflorescence with reduced bracts, and *Fargesia* for its short-necked pachymorph rhizome (Stapleton 1994). Moreover, most species of *Borinda* were transferred from *Fargesia* (Stapleton 1998, 2006, 2021). Although morphological differentiation of rhizome and inflorescence has been used to distinguish these genera, the intermediate state of rhizome and inflorescence between them makes the genus delimitation very ambiguous. As a result, the bamboo accounts of the “Flora of China” recognize two genera, i.e., *Fargesia* and *Yushania* (Li et al. 2006).

In our recent molecular analyses of this taxonomically difficult group based on the double digest-restriction-site associated DNA sequencing (ddRAD) analyses (Ye et al. 2019), *Yushania* was resolved as a well-supported monophyletic lineage, demonstrating the phylogenetic importance of the rhizome type. If considering the rhizome type alone, *Fargesiayunnanensis* Hsueh & T.P. Yi needs to be transferred into *Yushania* for its generally long rhizome neck (12–35 cm). Actually, this species was nested in the ‘Fargesia1’ clade in the analysis of Ye et al. (2019) with high support. Therefore, more information on inflorescence knowledge should be provided for the delimitation of *Yushania*.

*Fargesia* was resolved as a polyphyly (Wang et al. 2017; Zhang et al. 2019; Zhou et al. 2019; Zhou et al. 2020; Ye et al. 2021a) and divided into several clades with high support in the recently ddRAD analyses (Ye et al. 2019). Stapleton (2021) transferred species in the ‘Fargesia3’ + ‘Fargesia4’ + *F.angustissima* clade of Ye et al. (2019) and several species sampled by Zhang et al. (2019) into *Borinda* based on the molecular phylogenetics and some floral and vegetative characteristics. Concurrently, Stapleton (2021) considered that *Fargesia* s. s. possesses tightly unilateral racemes and only distributed along the Qinling Mountains. In this case, most of the species originally described in *Fargesia* could not be retained in this genus. Nevertheless, several species which were transferred into *Borinda* shared the floral characteristics of *Fargesia* s. s., with raceme enclosing by spathe-like sheaths and protruding from unilateral side, such as *F.edulis* Hsueh & T.P. Yi and *F.adpressa* T.P. Yi (Li et al. 2006; Shi et al. 2022). This indicates that limited reproductive characters cannot distinguish *Borinda* from *Fargesia* appropriately. Therefore, more knowledge of reproductive features should be provided to improve our understanding of the relationship of *Borinda*, *Fargesia* and *Yushania*.

In recent field surveys, we collected the floral and vegetative specimens for two bamboo species. A supplementary description of the inflorescence of these two species is presented here, providing new information on the delimitation of alpine bamboos.

## ﻿Materials and methods

We collected two specimens with both floral and vegetative organs during our field work in Yunnan (*YXY2020023*) and Sichuan (*WL2021001*), China. Morphological studies were based on the living individuals in the field, specimens, and literature (Yi 1985, 1988a, b; Keng and Wang 1996; Li et al. 2006; Shi et al. 2022). Flowering and fruit materials were dissected under an OLYMPUS DP80 digital microscope at Germplasm Bank of Wild Species of the Kunming Institute of Botany. The morphological terminology follows McClure (McClure 1966).

## ﻿Results

According to our observation and comparison of the type specimens and original literature (Yi 1985, 1988b) and bamboo accounts of “Flora Reipublicae Popularis Sinicae” (Keng and Wang 1996), “Flora of China” (Li et al. 2006) and “Illustrated Flora of Bambusoideae in China” (Shi et al. 2022), we identified specimen *YXY2020023* to be *Yushaniapauciramificans* T.P. Yi based on paniculate inflorescence on terminating leafy branches without spathes subtending, pachymorph rhizomes with long neck (20–50 cm in length), culms 2–3.2 m, internodes terete, branches 1–3 at lower nodes, 5–6 at upper, culm sheaths cartilaginous, with erect gray setae and absent auricles. *WL2021001* was identified to be *Fargesiaangustissima* T.P. Yi according to panicles on terminating leafy branches subtended by slightly expanded bracts, pachymorph rhizomes with short neck (2–5 cm in length), unicaespitose culms with fine ridged internodes, culm node less prominent than sheath scar, culm sheaths persistent, which were longer than internodes, narrowly triangular, apically papery, linear, and narrowed for distal 1/3–1/2 of length, sparsely brown setulose, leaf blade abaxially proximally white-gray pubescent. All voucher specimens were deposited at Kunming Institute of Botany, Chinese Academy of Sciences (KUN), and epitype of these two species are also designated here (Turland et al. 2018).

### ﻿Taxonomic treatment

#### ﻿ Fargesia angustissima

Taxon classificationPlantaePoalesPoaceae

T.P. Yi

3724B35B-BA71-52D0-91CA-A2E250645404

**Figure 1.**
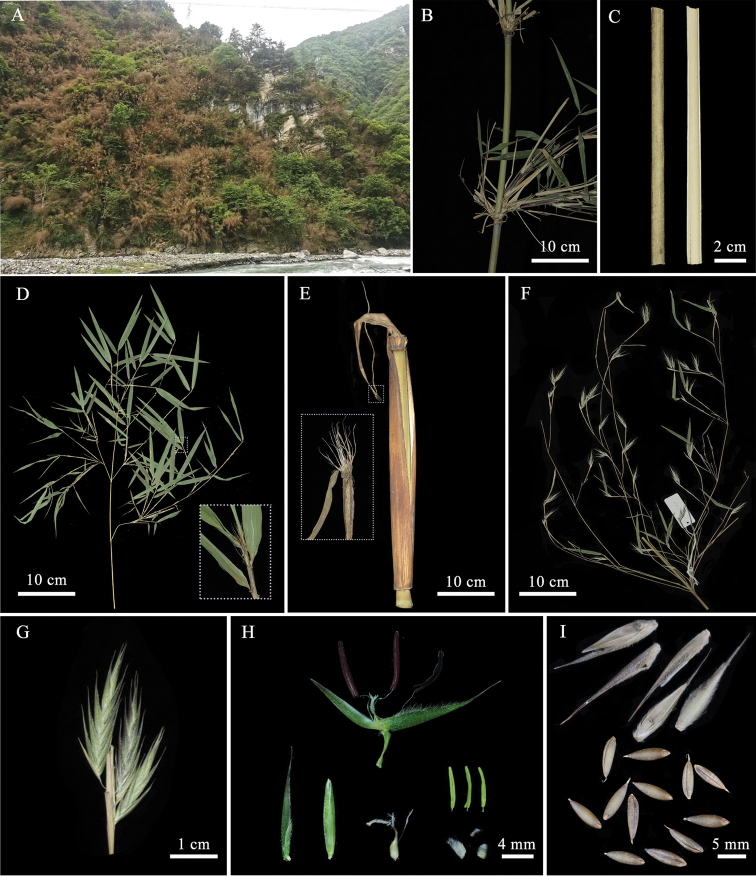
*Fargesiaangustissima* T.P. Yi **A** habitat, showing flowering population **B** branches and internode **C** clum **D** branchlet, showing foliage leaves **E** culm sheath **F** flowering branches **G** inflorescence **H** anatomy of florets **I** fruit.

“油竹子” (You Zhu Zi) Fig. 1

Fargesia angustissima T.P. Yi in J. Bamboo Res. 4(2): 21–22. pl. 4. 1985; Keng f. & Z. P. Wang, Fl. Reippubl. Poppularis. Sin. 9(1): 437. pl. 50, 1–8. 1996; D. Z. Li and Stapleton in Z. Y. Wu, P. H. Raven & D. Y. Hong, Fl. China 22: 85–86. 2006. L. B. Zhang in C. Y. Wu, P. H. Raven & H. Y. Hong, Fl. China Illustr. 22: 110. pl. 110: 1–7. 2007. ‘Type’: China. Sichuan: Wenchuan County, Genda Township, 1550 m alt., live on limestone slope, 22 Sept. 1974, *T.P. Yi 74450* (holotype, SCFI!); ibid., 31°4.27'N, 103°19.64'E, 1434 m alt., 23 Dec. 2021, *WL2021001* (epitype designated here, KUN, 1546903!).
≡ Yushaniaferaxsubsp.angustissima (T.P. Yi) Demoly in Bambou. Bull. A. E. B., Sect. France. 46: 8. 2005.
≡ Borindaangustissima (T.P. Yi) Stapleton in Sida. 22(1): 332, 2006.

##### Description.

Culms densely unicaespitose, 4–7 m tall, 1–2 cm in diameter; internodes terete, 28–35 cm long, glabrous, initially white powdery, longitudinal ribs very prominent; culm walls 1.5–2.5 mm thick; sheath scars prominent. Buds oblong. Branches 5–10 per node, slender. Culm sheaths persistent or gradually deciduous, much longer than internodes, distantly papery and narrowly banded, abaxially sparsely brown setulose, margins initially densely ciliate; auricles absent; oral setae 3–5, 5–7 mm long; ligules ca. 1 mm tall; blade reflexed, linear, narrower than apex of sheath, margins usually serrulate, readily deciduous. Foliage leaves 3–5 per branchlet; auricles absent; oral setae 5–8, 2–3 mm long; ligules convex, ca. 0.5 mm tall, external ligule pubescent; blades (1.7) 3.4–9.5 × 0.3–0.7 cm, narrowly lanceolate, abaxially proximally pubescent, second veins 2 (3) pairs, transverse veins distinct.

Flowering branches 18–60 cm long, with secondary branches; raceme composed of 1–3 spikelets, open to dense, flowering branchlet with terminal leaves 1–2, gradually deciduous, subtended by slightly inflated foliage-leaf-like sheaths, initially stretching out from one side of the sheaths; axes terete, glabrous, usually with a bract at the base of pedicels, bract lanceolate, 5 mm long. Spikelet purple-green to dark purple, 2–4 cm long, 7–10 mm wide, clustered closely; pedicels slender, curved or undulate, glabrous, 7–15 mm long; florets 2–8, 1.5–2 cm long; rachilla internode 2 mm long, expanded and pubescent at apex. Glumes 2, papery, apex acuminate, the first one narrowly lanceolate, 9–12 mm long, 2 mm wide, pubescent, apically awned, ca. 3 mm long; the second one ovoid-lanceolate, 11–15 mm long, ca. 2 mm wide, pubescent, apically awned, ca. 5 mm long. Lemma papery, ovoid-lanceolate, 15–19 mm long, 4–5 mm wide, abaxially scabrous, densely white setose, apically awned, 5–6 mm long; palea shorter than lemma, thinly papery, 5–10 mm long, 2-keeled, sulcate between keels, upper part of keels ciliate, apex obtuse. Lodicules 3, membranous, transparent, elliptical-triangular, margins ciliate. Stamens 3, filaments free, anthers yellow, gradually dark purple, ovary long-ovoid, glabrous. Stigmas 3, plumose. Caryopses oblong, dark brown, ventrally grooved, 6–9 mm long, ca. 1–2 mm in diameter, glabrous, apex with persistent style.

##### Phenology.

New shoots May to August. Flowering December to April; fruiting May to June.

##### Distribution and habitat.

*Fargesiaangustissima* is known from Dujiangyan, Wenchuan and Chongzhou of western Sichuan, and Beichuan and Pingwu of northwestern Sichuan, and mainly occurs on the steep limestone slope or along the stream at an elevation of 800–1800 m.

##### Additional specimens examined.

China. Sichuan: Beichuan County, Caijiaping, near Xiaozhaizigou Nature Reserve, 09 Nov. 2017, Y. X. Zhang 17142 (KUN!).

#### ﻿ Yushania pauciramificans

Taxon classificationPlantaePoalesPoaceae

T.P. Yi

C27908B3-C4C3-5E5C-9A4A-B2DAA01FCE94

**Figure 2.**
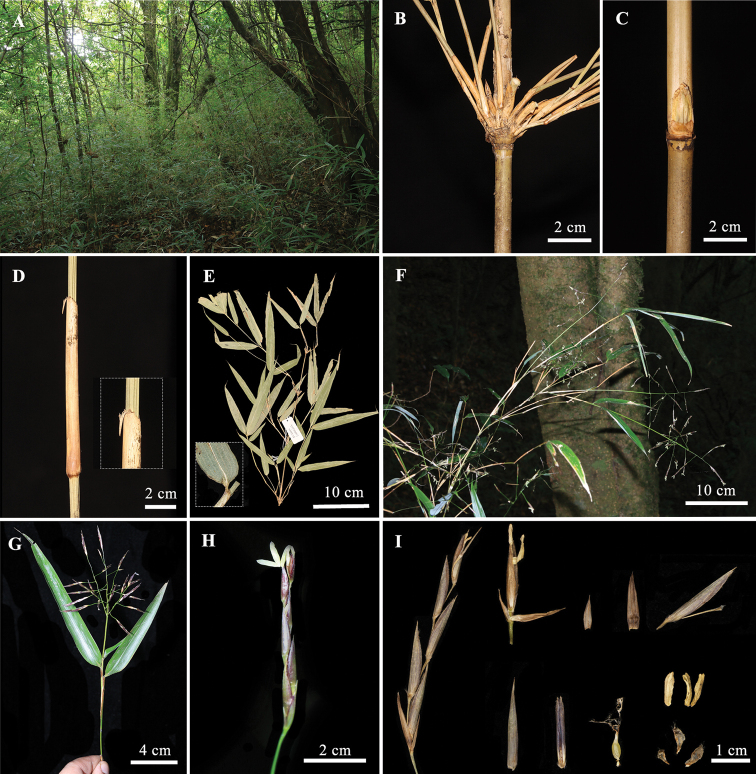
*Yushaniapauciramificans* T.P. Yi **A** habitat **B** branches **C** bud **D** culm sheath **E** branchlet, showing foliage leaves **F** flowering branches **G** inflorescence **H** spikelet **I** anatomy of florets.

“少枝玉山竹” (Shao Zhi Yu Shan Zhu) Fig. 2

Yushania pauciramificans T.P. Yi in Bull. Bot. Res. 8(4): 71–73. pl. 5. 1988; Keng f. & Z. P. Wang, Fl. Reippubl. Poppularis. Sin. 9(1): 547. pl. 164, 6–8. 1996; D. Z. Li and Stapleton in Z. Y. Wu, P. H. Raven & D. Y. Hong, Fl. China 22: 72. 2006. L. B. Zhang in C. Y. Wu, P. H. Raven & H. Y. Hong, Fl. China Illustr. 22: 96. pl. 96: 6–8. 2007. ‘Type’: China. Yunnan: Xinping County, Ailao Mountain, Liangshan, 2510 m alt., under forest, 1 Sept. 1986, *T.P. Yi 86237* (holotype, SCFI!); ibid., Gasa Town, 23°57'N, 101°33.90'E, 2257 m alt., 28 May 2020, *YXY2020023* (epitype designated here, KUN, 1546904!).

##### Description.

Culms diffuse, 1.5–3.5 m tall, 0.6–1.2 cm in diameter; internodes terete, 15–27 cm long, initially with a white powdery ring below nodes, glabrous; culm walls 2.5–3.5 mm thick, cavity small; nodes weakly prominent; sheath scar obviously prominent, woody. Branches 1–3 at lower nodes, ca. 5 at upper. Culm sheaths persistent, triangularly oblong, 2/5–1/2 as long as internodes, cartilaginous, gray setose abaxially, margins densely setose; auricles absent; oral setae 2–4, erect, deciduous; ligule 1–1.5 mm tall, glabrous; blades linear lanceolate, glabrous, reflexed. Foliage leaves 2–5 per branchlet; sheath margins glabrous; auricles absent; oral setae 5–7, slightly curved; ligule 0.5–1 mm tall; blades 5.2–16 × 1.1–2.8 cm, lanceolate or elliptic-lanceolate, base broadly cuneate or rounded, glabrous, secondary veins 4–6 pairs, transverse veins distinct.

Flowering branches 6–22 cm long, lower nodes with secondary flowering branchlets; inflorescence open, paniculate, terminal on leafy branches, composed of 5–15 spikelets, axilla with tuberculate glands, subtended by a small bract; axes terete, 2–10 cm long, glabrous. Spikelet dark purple, 3–6 cm long; pedicels slender, 1.3–3 cm long, usually slightly sinuous, glabrous; florets 2–5, 1.2–3 cm long, cylindrical, apical floret sterile and tubulose; rachilla internodes slightly flattened, ca. 5 mm long, gray white pubescent, apex more densely, margins gray white ciliate. Glumes 2, apically awned, ca. 1 mm, the first one narrowly lanceolate, 4–7 mm long, ca. 1 mm wide, distally white pubescent; the second one ovoid-lanceolate, 6–10 mm long, 1.5–2 mm wide, distally white pubescent. Lemma mucronate, papery, 7–10 mm long, 3–4 mm wide, densely white setose, apically awned, ca. 1 mm; palea slightly shorter than lemma, thinly papery, 6–9 mm long, 2-keeled, densely pubescent, apex obtuse, 2-cleft. Lodicules 3, membranous, transparent, elliptical-triangular, margins ciliate. Stamens 3, filaments free, anthers yellow, ovary long-ovoid, glabrous. Pistil short, stigmas 2, plumose. Fruits unknown.

##### Phenology.

New shoots August. Flowering April to June.

##### Distribution and habitat.

*Yushaniapauciramificans* is known from Xinping, south-central Yunnan, mainly distributed in the evergreen broadleaved forest at an elevation of 2250–2500 m.

## ﻿Discussion

**Figure 3.**
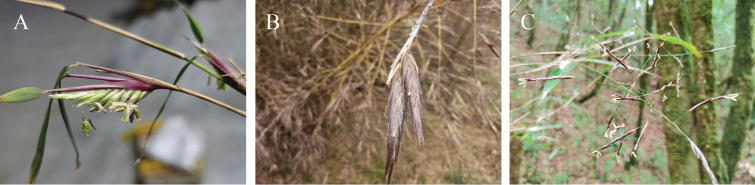
Comparison of inflorescence **A***Fargesiafuniushanensis* T.P. Yi **B***Fargesiaangustissima* T.P. Yi **C***Yushaniapauciramificans* T.P. Yi.

Reproductive features play an important role in the delimitation of the genera of alpine bamboos, and can improve our understanding of the relationship of *Borinda*, *Fargesia* and *Yushania*. Although *Fargesiaangustissima* was recombined into *Borinda* in the analysis of Stapleton (2006), its inflorescence characters provide new insight in its delimitation. According to the description, the leaf sheath underneath the inflorescence of *F.angustissima* is inflated but smaller than the spathe of some species of *Fargesia* s. s., such as *F.funiushanensis* T.P. Yi, *F.qinlingensis* T.P. Yi & J. X. Shao, while more similar to those with spikelets stretching out from one side of sheaths and arranging relatively loosely (Zhang and Ren 2016). These characteristics indicate that the inflorescence of *F.angustissima* is in a transitional state between compressed and open ones (Fig. 3). Moreover, *F.angustissima* possesses an independent position on the molecular phylogenetic trees and has a special habitat (Ye et al. 2019; Shi et al. 2022). The florets, phylogenetic position, and habitat of *F.angustissima* imply that it is different from other species of *Fargesia* and could be treated as a new genus, albeit following a narrower genus concept. Additionally, as currently circumscribed, *Borinda* is a paraphyletic group without distinct synapomorphies despite some hair-like vegetative morphology and gene flow occurring frequently between it and Fargesia1 clade (Ye et al. 2019; Stapleton 2021; Ye et al. 2021a). Considering the variable vegetative characters and insufficient reproductive features, any new combination of these alpine bamboos should be made cautiously, especially when extensive gene flow exists. Thus, we support the “Flora of China” in adopting a broadly-defined *Fargesia* s. l., rather than *Borinda* to minimize nomenclatural change.

The flowering branches and flowers of *Yushaniapauciramificans* are similar to those of species of *Yushania*, both have similar paniculate inflorescence, axilla with tuberculate glands, ciliate rachilla internodes and florets. Combined with the reduced gene flow between *Yushania* and *Fargesia* revealed by D-statistic tests, the inflorescence state of *Y.pauciramificans* supports the monophyly of *Yushania* further (Ye et al. 2019).

## Supplementary Material

XML Treatment for Fargesia angustissima

XML Treatment for Yushania pauciramificans
